# The Relationship Between Childhood Maltreatment and Insomnia in Depressed Adolescents: The Mediating Role of Rumination

**DOI:** 10.1002/brb3.70554

**Published:** 2025-05-13

**Authors:** Aiping Wang, Zhaojuan Xu, Caifeng Liu, Lingxia Cao, Feifei Wang

**Affiliations:** ^1^ Department of Clinical Psychology, Shandong Mental Health Center Shandong University Jinan Shandong China; ^2^ The Second Hospital of Shandong University Jinan Shandong China

**Keywords:** adolescents | childhood maltreatment | insomnia | major depressive disorder | rumination

## Abstract

**Background:**

There exists a prevalence of insomnia in depressed adolescents. Childhood maltreatment is a major life event which may intensify its severity. However, studies on the relationship between insomnia and childhood maltreatment and the mediating role of rumination are scarce.

**Methods:**

The study utilized a cross‐sectional study design with 88 adolescents diagnosed with major depressive disorder. Childhood maltreatment, rumination, and insomnia were measured by self‐reported questionnaires which included Child Psychological Abuse and Neglect Scale, Rumination Response Scale, and Insomnia Severity Index. Multiple linear regressions were employed for data analysis after controlling for confounders.

**Results:**

Adolescents with severe childhood neglect reported higher levels of insomnia. Childhood abuse was positively associated with insomnia, but their association was insignificant. Symptom rumination and reflective pondering could indirectly mediate the association between neglect and insomnia.

**Conclusion:**

The association between childhood maltreatment and insomnia symptoms in depressed adolescents was mediated by rumination. Collaboration with psychotherapists who are experts in psychotherapies, like cognition or mindfulness‐based intervention, may be an effective way of releasing the adverse effects of childhood maltreatment on insomnia.

## Graphical abstract

We suggested an early screening and prevention of child maltreatment to prevent the onset of insomnia symptoms in adolescents with major depressive disorders. Special attention should be given to the child neglect, which is often ignored and difficult to detect in practice. Moreover, this study found symptom rumination and reflective pondering may mediate the association between childhood maltreatment and insomnia, which may be intervention targets to reduce the adverse effects of child neglect on insomnia symptoms.

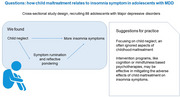

## 1 Background

Adolescents are vulnerable for major depressive disorder (MDD), which affects 8% of adolescents around the world (Shorey et al. 2022). It is associated with academic difficulties, poor interpersonal relationships, and even suicide in adolescence and may have long‐lasting effects in adulthood (Clayborne et al. 2019; Keenan‐Miller et al. 2007). Strikingly, MDD among adolescents is often neglected due to the misattributions to normal moods or stages adolescents have to go through, with only 50% of adolescents diagnosed before they get into adulthood (Mullen 2018). Moreover, adolescents under the treatment may still be at a high risk of relapse (Mullen 2018). This makes it important to look for new treatment targets and focus on earlier risk factors of MDD in adolescents.

Insomnia is considered a symptom of depression for a long time; however, recently, it is reconsidered an independent disorder, a comorbidity with depression (Sweetman et al. 2021). A recent meta‐analysis that included longitudinal studies even found insomnia may be a risk factor for subsequent depression (odds ratio = 2.10) (Baglioni et al. 2011). Randomized controlled trials confirmed these findings by reporting that intervening on insomnia could effectively reduce depressive symptoms in patients (Gebara et al. 2018). These findings together suggested that insomnia symptoms may be a target to manage depression symptoms among adolescents with MDD.

From a life course perspective, childhood adverse experience is regarded as a distal factor for psychological symptoms in later life. Two meta‐analyses found that childhood maltreatment was associated with both depression and insomnia in children or adolescents (Infurna et al. 2016; Schønning et al. 2022). Taylor et al. (2016) confirmed the role of childhood maltreatment in insomnia in the army and underscored the importance of physical neglect (odds ratio = 1.33), which is often neglected in current studies. However, these studies are not specifically designed for adolescents with MDD. Factors through which childhood maltreatment influences insomnia symptoms in adolescents with MDD are also not explored in this study.

According to the cognition model of insomnia, rumination is a precipitating risk factor as it leads to biased attention and unpleasant intrusive thoughts that disrupt sleep‐related cues (Harvey 2002). This model is supported by empirical studies. For example, Rachel et al. found that 87% of adolescents with sleep problems had excessive thinking related to threat‐related experiences (Hiller et al. 2014). A review found that those exposed to childhood maltreatment tend to repeat negative thinking (rumination) (Mansueto et al. 2021). Some studies also suggested that those who have experienced childhood maltreatment were more likely to have rumination (Baer et al. 2012; Zielinski et al. 2015). Taking together, these findings suggested that rumination may mediate the association between childhood maltreatment and insomnia symptoms in depressed adolescents. However, a formal mediation analysis remains scarce.

Therefore, this study focused on depressed adolescents and aimed at (1) exploring the relationships between each type of childhood maltreatment and insomnia, and (2) examining whether rumination could mediate the relationship between childhood maltreatment and insomnia. In line with previous studies, we hypothesize that (1) different types of childhood maltreatment have different associations with insomnia in depressed adolescents, and (2) rumination could mediate their associations.

## 2 Methods

### 2.1 Participants

This study recruited adolescents from a tertiary psychiatry hospital in Shandong Province, China between September 2022 and November 2022. The participants were included in the study if (1) they were aged between 12 and 19 years old, (2) they were diagnosed with MDD by psychiatrists according to the Diagnostic and Statistical Manual of Mental Disorder, Fifth Edition (DSM‐5) criterion, (3) they could write and speak Chinese, and (4) parental consent was given to participate in the study. We excluded those who were diagnosed with bipolar disorder, schizophrenia, or other mental disorders.

This study conformed to the Declaration of Helsinki and was approved by the Ethical Committee in the Shandong Mental Health Center (2022‐100). All adolescents and their parents gave their informed consent prior to their inclusion in the study.

### 2.2 Measures

We collected data on demographics and lifestyle. The demographic information included adolescents’ age, sex, grade, academic performance, details about parents’ educational status, and their relationship status. Lifestyle included smoking and alcohol drink status.

Insomnia was assessed using the Insomnia Severity Index (ISI) (Morin et al. 2011). ISI consists of seven items, and each item is rated from 0 to 3. The summed score of ISI indicates the severity of insomnia, with higher scores indicating more severe insomnia. It is evidenced as valid and reliable among Chinese adolescents (Chung et al. 2011). Its Cronbach's alpha coefficient in this study was 0.783.

Childhood maltreatment was measured by the Child Psychological Abuse and Neglect Scale (CPANS) (Deng et al. 2007). It contains 31 items, and each item is rated from 0 (never) to 4 (always). CPANS comprises two subscales (abuse and neglect), and each subscale includes three subdomains (abuse: scold, threaten, intervene; neglect: emotional neglect, educational neglect, supervision neglect). CPANS is reliable and valid for the Chinese population (Deng et al. 2007). In this study, the Cronbach's alpha coefficient of each subscale and subdomain ranged from 0.683 to 0.889.

Rumination was measured by the Chinese version of the Rumination Response Scale (RRS). It consists of 21 items and 3 factors (symptom rumination, brooding, and reflective pondering). Each item is rated from 1 (*never*) to 4 (*always*). The summed score for each factor indicated the level of rumination: higher scores indicated higher levels of rumination. Its validity and reliability have been demonstrated in the Chinese population (Liu et al. 2022). Its Cronbach's alpha coefficient in this study was 0.924.

### 2.3 Statistical Analysis

Participants’ data were described using means or frequency. A multiple linear regression model was employed to explore the relationship between each type of childhood maltreatment and insomnia after controlling for adolescents’ age, sex, grade, parents’ educational status, and whether they had siblings. The relationship between insomnia and symptoms of depression was also explored in the multiple linear regression models, after controlling for covariates. Further, we used the mediation model to examine the mediating role of rumination in the association between childhood maltreatment and insomnia. In the mediation model, bias‐corrected bootstrapped method based on 2000 samples was used. The confidence interval (CI) level and significance level were set at 95% and 5%, respectively. In the sensitivity analysis, we replicate the mediation analysis only in females. In the post hoc power analysis for mediation analysis (Schoemann et al. 2017), our study reached the power of 78%. Statistical analysis was performed using Statistical Package for Social Sciences (SPSS 26.0) and Analysis of Moment Structure (AMOS 26.0). The two‐tailed test with *p* value <0.5 was set.

## 3 Results

### 3.1 Characteristics of Study Participants

The study participants were 88 adolescents. Their mean age was 15.2 ± 1.6 years old. Overall, 83% of the participants were female. Half of them were in secondary school or primary school. Approximately 70% of the participants belonged to a family of two or more children. Ten percent of adolescents came from the family of single parents. About 70% of adolescents witnessed quarrels between parents. The education level of approximately 66% of parents was lower than that of high school. About 70% of them are ranked in the top 50% in academic performance. No physically diagnosed disease was seen in 91% of the adolescents. More details are described in Table 1.

**TABLE 1:** Characteristics of adolescents in the study (*n* = 88).

Variables	Mean (standard deviation)/Frequency (percentage)
Age (years)	15.2 (1.6)
Sex	
Female	73 (83.0%)
Male	15 (17.0%)
Grade	
Secondary school or lower	46 (52.3%)
High school	23 (26.1%)
College	14 (15.9%)
University or more	5 (5.7%)
One child family	
Yes	25 (28.4%)
No	63 (71.6%)
Parental relationship status	
Good marital relation	19 (21.6%)
Sometimes having quarrels	35 (39.8%)
Often having quarrels	25 (28.4%)
Divorced/Separate/Passed away	9 (10.2%)
Parental education status	
Primary school	7 (8.0%)
Secondary school	33 (37.5%)
High school	18 (20.5%)
College	14 (15.9%)
Graduate	8 (9.1%)
Undergraduate	8 (9.1%)
Rank in academic performance	
Top 5%	17 (19.3%)
5%–25%	17 (19.3%)
25%–50%	23 (26.1%)
Behind 50%	31 (35.2%)
Having diagnosed physical disease	
Healthy	80 (90.9%)
Having disease history	8 (9.1%)

### 3.2 Insomnia, Rumination, and Depression Symptoms in Adolescents

As shown in Table 2, the average score obtained for ISI was 4.3 (4.0). The average score for the depression subscale of DASS was 11.7 (6.8); for neglect subscale of CPANS, it was 23.9 (13.6), and for abuse subscale of CPANS, it was 23.8 (11.9). The average score for the symptom rumination subscale of RRS was 32.8 (8.9); for the reflective pondering subscale of RRS, it was 12.9 (3.5), and for the brooding subscale of RRS, it was 14.4 (3.3).

**TABLE 2:** The distribution of depression and child maltreatment in the study (*n* = 88).

Variables	Mean (standard deviation)
Depression score in DASS	11.7 (6.8)
Score in ISI	4.3 (4.0)
Neglect score in CPANS	23.9 (13.6)
Abuse score in CPANS	23.8 (11.9)
Score in RRS	
Symptom rumination	32.8 (8.9)
Reflective pondering	12.9 (3.5)
Brooding	14.4 (3.3)

Abbreviations: CPANS, child psychological abuse and neglect scale; DASS, depression, anxiety and stress scale; ISI, insomnia severity index; RRS, rumination response scale.

The relationship between insomnia and depression was significant (*B* = 0.773, 95%CI = 0.453–1.094, *p *< 0.001), after controlling for adolescents’ age, sex, grade, parents’ educational status, and whether they had siblings (Table 3). After controlling for the covariates, we found adolescents’ score in total and for each subscale of neglect to be positively associated with their score in ISI (*B* = 0.091–0.305). The relationship between abuse and insomnia was found to be insignificant (all *p *> 0.05) (Table 3).

**TABLE 3:** Multiple linear regressions for the association between maltreatment experiences and insomnia problem in adolescents with major depressive disorder (*n* = 88).

Variables	Insomnia			
	*B* value	95% confidence interval	*p* value	
Maltreatment experiences				
Neglect in total	0.091	0.027–0.155	0.006	
Emotional neglect	0.169	0.040–0.297	0.011	
Educational neglect	0.305	0.086–0.523	0.007	
Physical/Supervision neglect	0.235	0.022–0.449	0.031	
Abuse in total	0.065	−0.009 to 0.139	0.085	
Criticism	0.162	−0.052 to 0.377	0.136	
Threatening	0.154	−0.066 to 0.373	0.168	
Intervening	0.162	−0.018 to 0.341	0.077	

*Note*: Adjusted variables include: adolescents’ age, sex, grade, parents’ education status, whether had sisters or brothers.

### 3.3 Mediating Role of Rumination in the Association Between Neglect and Insomnia

**FIGURE 1:**
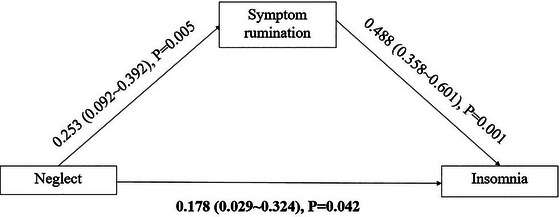
The standardized regression coefficients for the mediating role of symptom rumination in the association between neglect and insomnia.

**FIGURE 2:**
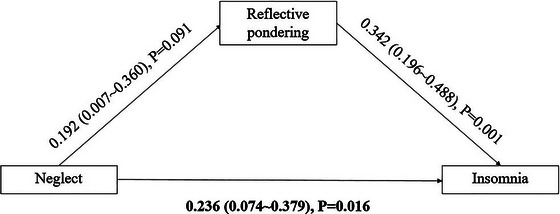
The standardized regression coefficients for the mediating role of reflective pondering in the association between neglect and insomnia.

In the mediating model, we found that symptom rumination (standardized indirect effect size = 0.123, 95%CI = 0.038–0.240) (Figure 1) and reflective pondering (standardized indirect effect size = 0.066, 95%CI = 0.001–0.164) (Figure 2) mediated the association between neglect and insomnia. Symptom rumination accounted for 16.82% of the total effect, and reflective pondering accounted for 7.45% of the total effect. The mediating effect of brooding was not observed (standardized indirect effect size = 0.022, 95%CI = −0.035–0.097).

In the sensitivity analysis, we only found the significant mediating role of symptom rumination (standardized indirect effect size = 0.141, 95%CI = 0.036–0.268) in girls. We did not observe the mediating role of reflective pondering (standardized indirect effect size = 0.060, 95%CI = −0.022 to 0.170) and brooding (standardized indirect effect size = 0.021, 95%CI = −0.048 to 0.110).

## 4 Discussion

This is the first study to investigate the relationship between each type of childhood maltreatment and insomnia in depressed adolescents. We found childhood neglect to be significantly related to insomnia. Depressed adolescents with experiences of childhood neglect were more likely to report more severe levels of insomnia. Although childhood abuse is positively associated with insomnia, the association is not significant. We also found that symptom rumination and reflective pondering could mediate the relationship between childhood neglect and insomnia in depressed adolescents.

In this study, the average score of ISI in depressed adolescents was 4.3, which was higher than the ISI score reported in Chinese general population (3.1) (Wang et al. 2021). Our results also supported previous findings about the positive association between insomnia and the severity of depressive symptoms in adolescents (Perlis et al. 1997; Sweetman et al. 2021). We advise that healthcare staff dedicate attention to insomnia in depressed adolescents, considering the critical role it plays in the progression and relapse of symptoms of depression in patients diagnosed with MDD.

This study observed a significant association between childhood maltreatment and insomnia, which is consistent with Reffi et al. (2022)’s study. Notably, Reffi's study solely focuses on child abuse. Another important type of childhood maltreatment is neglect. Child neglect is the most common type of maltreatment and is characterized by “omission.” Neglected children are deprived of access to basic care and emotional support from caregivers. We add this research gap and highlight the even more important role of child neglect than child abuse in the insomnia symptoms among adolescents with MDD.

Previous studies linked childhood experiences with ruminative tendencies in adulthood (Baer et al. 2012; Sarin and Nolen‐Hoeksema 2010; Spasojević and Alloy 2002). Adolescents in neglectful environments have little opportunity to learn adaptive emotion regulation skills. They are prone to ruminate on depressive symptoms and reflectively ponder when facing stressful life events. Besides, their lack of knowledge on how to deal with sleep problems puts them at a higher risk of developing insomnia. Therefore, we highlight the important role of rumination in the association between childhood maltreatment, especially for child neglect, and insomnia symptoms in adolescents with MDD. However, limited by our cross‐sectional study design, we cannot exclude the possible reverse association between insomnia and rumination. We suggested that future studies would benefit from a prospective study design.

The findings of our study have important implications for clinical practice in pediatric psychiatric symptoms. Health workers, who are advocates for patients and their families, play an important role in both child protection services in primary care and psychological care in clinical settings. In primary care, health workers can contribute a lot to detect the occurrence of child maltreatment as early as possible in the community. This could timely prevent the adverse effects of child maltreatment on insomnia symptoms in adolescents with MDDs. Considering the intertwining association between insomnia and depression, it is important to reduce insomnia symptoms in adolescents with MDDs.

Clinical workers in psychiatric clinics should pay attention to rumination symptoms in adolescents with MDDs. Suitable psychotherapies, which target at reducing rumination, may be effective in reducing the adverse effects of childhood treatment in adolescents with MDDs. Collaboration among psychotherapists or psychiatrists can ensure that patients are undergoing adequate support from multiple facets.

### 4.1 Limitations

The study has several limitations. First, this is a cross‐sectional study. Although the model in the study was proposed by well‐accepted theory, we cannot rule out the reverse causality between variables. Second, childhood maltreatment in the current study was retrospectively collected by self‐reported measures. Hence, the possibility of recall bias must be considered when interpreting the findings. Third, the sample size in the current study is relatively small. Although we recruited a clinical sample that was vulnerable to insomnia, a larger sample size is needed to verify our results further. Studies with larger sample sizes and longitudinally collected data fromearly childhood will contribute to better understanding childhood contributors to insomnia and the role of rumination tendencies. Fourth, although we have controlled covariates in this study, there may still exist other unmeasured covariates, like parental mental health, and children's perceived social support from families and schools, which may bias our estimates. We advise these covariates should be taken into account infuture research.

## 5 Conclusion

This study found a close association between childhood maltreatment and insomnia in adolescents with MDDs. The significant role of one type of child maltreatment—child neglect—was stressed in this study. We suggest an early screening and prevention of child maltreatment to prevent the onset of insomnia symptoms in adolescents with MDDs. Special attention should be given to the child neglect issue, which is often ignored and difficult to detect in practice. Moreover, this study found symptom rumination and reflective pondering may mediate the association between childhood maltreatment and insomnia. This suggests that some available intervention programs, like cognition or mindfulness‐based psychotherapies, may be effective in mitigating the adverse effects of child maltreatment on insomnia symptoms in adolescents with MDDs.

## Author Contributions

Aiping Wang: Conceptualization, Data Curation, Formal analysis, Investigation, Methodology, Software, Visualization, Writing‐Original Draft Preparation. Zhaojuan Xu: Methodology, Validation, Writing‐Original Draft Preparation. Caifeng Liu: Methodology, Validation, Writing‐Review & Editing, Supervision, Project Administration, Resources, Conceptualization. Lingxia Cao: Data Curation, Investigation. Feifei Wang: Investigation, Data Curation.

## Conflicts of Interest

The authors declare no conflicts of interest.

### Peer Review

The peer review history for this article is available at https://publons.com/publon/10.1002/brb3.70554

## Data Availability

Data are available on request from the corresponding author.
